# Higher rates of metabolic syndrome among women taking zidovudine as compared to tenofovir in rural Africa: preliminary data from the CART-1 study

**DOI:** 10.7448/IAS.17.4.19552

**Published:** 2014-11-02

**Authors:** Niklaus Daniel Labhardt, Molisana Cheleboi, Olatunbosun Faturyiele, Mokete M Motlatsi, Karolin Pfeiffer, Thabo Ismael Lejone, Bernard Cerutti, Jürgen Muser, Ravi Shankar Gupta, Lutgarde Lynen, Christoph Hatz

**Affiliations:** 1 Department of Medical and Diagnostic Services, Swiss Tropical and Public Health Institute, Basel, Switzerland; 2 Laboratory Services, Seboche Hospital, Botha-Bothe, Lesotho; 3 SolidarMed, SolidarMed Lesotho, Thaba-Tseka, Lesotho; 4 SolidarMed, SolidarMed Switzerland, Lucerne, Switzerland; 5 Medical Faculty, University of Geneva, Geneva, Switzerland; 6 Central Laboratory, University Hospitals Basel-Land, Liestal, Switzerland; 7 Ministry of Health of Lesotho, DHMT Butha-Buthe, Lesotho; 8 Department of Clinical Sciences, Institute of Tropical Medicine, Antwerp, Belgium

## Abstract

**Introduction:**

Due to its side effects stavudine (D4T) has been replaced by zidovudine (AZT) and tenofovir (TDF) in most low- and middle-income countries (LMICs). In 2014 about 38% of adult first-line regimens contain AZT and 62% TDF [1]. Whereas the unfavourable metabolic outcomes of D4T in comparison to TDF have been described extensively, studies from LMICs comparing metabolic profiles between patients on AZT and TDF are scarce. Given the high number of patients in LMICs still taking AZT, data on their metabolic profile are needed. We present rates of metabolic syndrome (MS) in adult patients taking either AZT- or TDF-containing first-line, non-nucleoside reverse transcriptase (NNRTI)-based regimens.

**Materials and Methods:**

Data derived from a cross-sectional multi-disease screening conducted in ten facilities in two rural districts of Lesotho, Southern Africa [2]. Patients were eligible if aged ≥25 years and on NNRTI-containing first-line ART ≥6 months. The MS definition for Africa of the International Diabetes Federation was applied [3]. Assessed potential predictors for MS were age, time on ART, virologic suppression, body-mass index (BMI), alcohol consumption, wealth quintile, NNRTI (nevirapine (NVP) or Efavirenz (EFV)), history of previous D4T exposure and ART-backbone (AZT or TDF). Statistical analyses – stratified for sex – comprised univariate logistic regression for each predictor variable with subsequent construction of a multivariate model including all predictors with an association to MS at a significance level<0.1 in univariate analysis.

**Results:**

Out of 1026 patients, 660 (64.3%) were female. MS prevalence was 9.8% (95% CI 6.9–13.4) in men and 22.9% (19.7–26.3) in women. In women, aged ≥35 years, AZT-backbone, NVP-base, BMI ≥25kg/m2 and taking ART for ≥4.5 years were associated with MS in univariate analysis. In the multivariate model only AZT (adjusted odds-ratio: 2.2, 95% CI 1.4–3.6; p=0.001) and BMI ≥25kg/m2 (9.8; 2.8–34.1, p<0.001) were associated with MS. For men, age, higher wealth quintile, history of D4T exposure and BMI were associated with MS in univariate analysis. In the multivariate model only a BMI ≥25kg/m2 was associated with MS (8.9; 3.8–20.9, p<0.001).

**Conclusions:**

In rural Lesotho, Southern Africa, the use of AZT instead of TDF among women who are on ART for ≥6 months predisposes to the development of metabolic syndrome. Given that, still 38% of first-line regimens in LMIC contain AZT, this finding needs to be verified in other settings in Sub-Saharan Africa.
